# Phenolic-Enriched Collagen Fibrillar Coatings on Titanium Alloy to Promote Osteogenic Differentiation and Reduce Inflammation

**DOI:** 10.3390/ijms21176406

**Published:** 2020-09-03

**Authors:** Anna Mieszkowska, Harrison Beaumont, Laurine Martocq, Andrey Koptyug, Maria A. Surmeneva, Roman A. Surmenev, Javad Naderi, Timothy E.L. Douglas, Katarzyna A. Gurzawska-Comis

**Affiliations:** 1 Department of Microbiology, Faculty of Biochemistry, Biophysics and Biotechnology, Jagiellonian University, 31-007 Krakow, Poland; mieszkowska.an@gmail.com; 2 Oral Surgery Department, University of Birmingham, Birmingham B15 2TT, UK; k.a.gurzawska@bham.ac.uk; 3 Engineering Department, Lancaster University, Lancaster LA1 4YW, UK; h.beaumont2@lancaster.ac.uk (H.B.); t.douglas@lancaster.ac.uk (T.E.L.D.); 4 Department of Quality Technology, Mechanical Engineering & Mathematics, Mid Sweden University, 851 70 Östersund, Sweden; andrei.koptioug@miun.se; 5 Physical Materials Science and Composite Materials Centre, Research School of Chemistry & Applied Biomedical Sciences, National Research Tomsk Polytechnic University, 634050 Tomsk, Russia; surmenevamaria@mail.ru (M.A.S.); rsurmenev@mail.ru (R.A.S.); 6 Chemistry Department, Lancaster University, Lancaster LA1 4YW, UK; j.naderi@lancaster.ac.uk; 7 Materials Science Institute (MSI), Lancaster University, Lancaster LA1 4YW, UK

**Keywords:** collagen fibrils, coating, Ti6Al4V, phenolic, inflammation, osseointegration, osteoblast differentiation

## Abstract

The adsorption of biomolecules on biomaterial surfaces can promote their integration with surrounding tissue without changing their bulk properties. For biomaterials in bone reconstruction, the promotion of osteogenic differentiation and reduction of inflammation are desirable. Fibrillar coatings are interesting because of fibrils’ high surface area-volume ratio, aiding adsorption and adhesion. Fibrils also serve as a matrix for the immobilization of biomolecules with biological activity, such as the phenolic compound phloroglucinol (PG), the subunit of marine polyphenols. The aim of this work was to investigate the influence of PG coatings on fibroblast- and osteoblast-like cells to increase the osseointegration of titanium implants. Collagen fibril coatings, containing PG at low and high concentrations, were produced on titanium alloy (Ti6Al4V) scaffolds generated by additive manufacturing (AM). These coatings, especially PG-enriched coatings, reduced hydrophobicity and modulated the behavior of human osteosarcoma SaOS-2 and mouse embryonic fibroblast 3T3 cell lines. Both osteoblastic and fibroblastic cells spread and adhered well on PG-enriched coatings. Coatings significantly reduced the inflammatory response. Moreover, osteogenic differentiation was promoted by collagen coatings with a high PG concentration. Thus, the enrichment of collagen fibril coatings with PG is a promising strategy to improve Ti6Al4V implants for bone contact in orthopedics and dentistry and is worthy of further investigation.

## 1. Introduction

Titanium alloy (Ti6Al4V) is a “gold standard” material for load-bearing in orthopaedics and dentistry due its ability to withstand mechanical loading and resistance to corrosion. The long-term success of a titanium alloy implant is dependent on its stable fixation to the surrounding bone, which is dependent on osseointegration, i.e., the formation of a direct interface between an implant and bone, without intervening soft tissue. To achieve osseointegration, the promotion of osteoblastic differentiation and mitigation of inflammatory cell responses is necessary. A way to improve this process is by modifying the surface of implants with a coating.

Collagen fibril coatings can promote adhesion, proliferation and differentiation of rat calvarial osteoblasts [1,2] and osteoblast-like cells [3]. Protein coatings have been also reported to increase expression of genes characteristic for osteogenic differentiation [4]. Moreover, they have the advantage of a high surface/volume ratio that help fibrils to adhere to the surface. Studies have shown that fibrillar collagen coatings remain stable in physiological solutions [5] and have promoted bone formation around implants in vivo [6,7]. Collagen fibrils coatings are not only more stably anchored to substrates than coatings of tropocollagen (single collagen molecules or collagen triple helices) but also have been reported to improve cell synthesis of extracellular matrix more effectively [8]. Finally, they can act as matrices for the incorporation of biological molecules such as extracellular matrix components like glycosaminoglycans [9]. It is also possible to incorporate other biomolecules, including marine-derived polysaccharides [10], and proteins with biological activity including lactoferrin [11]. However, the incorporation of polyphenols and their phenolic subunits into collagen fibril coatings remains unexplored. One group of marine-derived polyphenols are phlorotannins, which are known to possess properties desirable for materials for bone contact, including the ability to promote mineralization [12] and exhibit antimicrobial properties especially against bacteria such as *Staphylococcus aureus (S. aureus)*, methicillin-resistant *S. aureus* (MRSA) and some fungi [12,13,14].

Phloroglucinol (PG), the phenolic subunit of phlorotannins, is thought to have properties such as anti-inflammatory effects [15]. Moreover, it reduces oxidative stress which is advantageous for bone regeneration. In fact, it has been reported that oxidative stress may hinder osteogenic differentiation [16,17,18,19] and stimulate bone resorption [20]. PG has exhibited antioxidant activity as a component of chitosan hydrogels in previous work [21]. Hence, one could hypothesize that PG could reduce the expression of genes responsible for inflammation.

This study focused on the influence of collagen fibrillar coatings containing PG adsorbed on Ti6Al4V samples on the inflammatory and osteogenic response of fibroblast-like cells and osteoblast-like cells in vitro. As osteoblasts are present in bone and fibroblasts are present in soft oral tissue, the behavior of these two types of cell is most relevant for biomaterials intended for bone contact in oral and maxillofacial surgery. We hypothesized that collagen fibrillar coatings enriched with PG could reduce the expression of genes specific for inflammation, such as interleukin-6 (*IL6*) and tumor necrosis factor-alpha (*TNFA*) in osteoblast-like cells (SaOS-2) and fibroblast-like cells (3T3 cell line), as well as promote the expression of genes specific for osteogenic differentiation, such as collagen type I alpha 1 chain (*COL1A1*) and bone gamma-carboxyglutamate protein (*BGLAP*) genes in osteoblast-like cells (SaOS-2).

To the best of our knowledge, phenolics such as PG have not yet been incorporated into collagen fibril-based coatings for biomaterial applications, and the effect of PG as a component of a coating on cell gene expression remains unexplored.

In this study, collagen fibril coatings were prepared by inducing fibril formation by neutralizing an acidic solution of tropocollagen solution. Fibril formation led, in turn, to formation of a collagen hydrogel, which was placed on the substrate surface to allow fibrils to adsorb onto the surface of the substrate and form a coating as in previous work [11]. PG was added to the tropocollagen solution prior to neutralization. This procedure is technically very simple and can be used to coat any implant geometry and topography, including the roughened Ti6Al4V surfaces used in this study.

## 2. Results and Discussion

### 2.1. Characterization of Collagen Hydrogels

**Figure 1:**
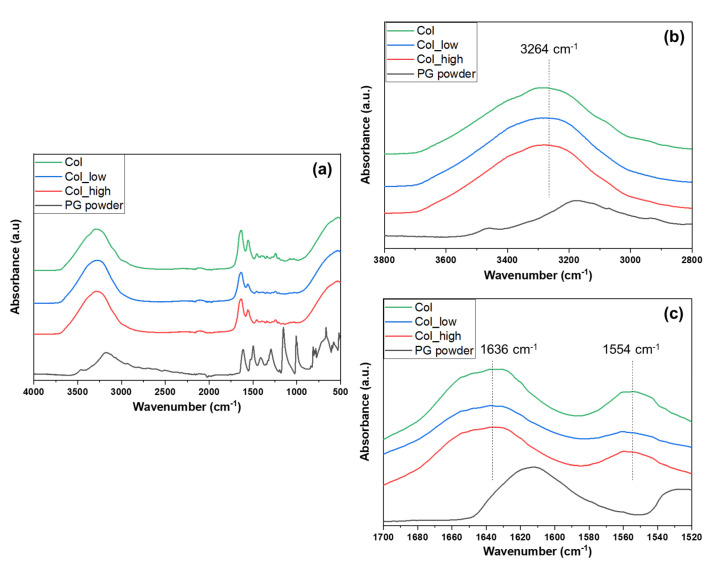
FTIR spectra of PG powder and collagen hydrogels containing no PG, 0.333 mg/mL and 1.0 mg/mL of PG solutions (**a**) in the 4000–500 cm^−1^, (**b**) in the 3800–2800 cm^−1^ and (**c**) in the 1700–1520 cm^−1^ wavenumber regions. Col: collagen hydrogel; Col_low: collagen hydrogel with a “low” PG concentration (0.333 mg/mL); Col_high: collagen hydrogel with a “high” PG concentration (1.0 mg/mL).

Fourier Transform Infrared (FTIR) spectroscopy analyses were carried out on collagen hydrogels containing no PG (Col), 0.333 mg/mL PG (Col_low) and 1.0 mg/mL PG (Col_high) (Figure 1). First, when PG concentration increased, the absorbance band related to C-O stretch from PG (1240 cm^−1^) increased as well, which demonstrates that collagen hydrogels are well enriched with PG. However, the O-H stretch feature could not be detected due to an overlapping with the NH_2_ groups found in collagen. In fact, an absorbance region was detected around 3260–3280 cm^−1^ across all samples which correlates to the NH_2_ bridges present in the primary structure of the protein. Furthermore, the band at around 1626–1635 cm^−1^ suggested the presence of the C=O stretch in the primary, secondary, and tertiary structure of collagen. The band in the region of 1550–1560 cm^−1^ indicated a N-H bend present in the secondary structure of a protein [22]. Furthermore, the addition of PG to the coating appeared to shift the position of the band wavelengths. Indeed, the band wavelengths of the N-H stretch increased slightly as the concentration on PG within the hydrogel also. Furthermore, the addition of PG to the hydrogels may have had an effect on C=O stretch (although this is not conclusive from the spectra) and reduced the wavelengths for N-H in-plane bend (for the highest PG concentration). This suggests a non-covalent interaction between PG and collagen. PG is the fundamental building block of phlorotannins; it has been reported that phlorotannins precipitate some proteins by covalent and non-covalent interactions [23]. Non-covalent interactions, determined mainly by hydrophobic interactions of aromatic groups of polyphenols with hydrophobic groups of the proteins, have been already reported for milk-derived protein and polyphenol by fluorescence quenching in previous studies [24,25,26]. Moreover, several studies have been performed on the influence of milk protein on tea polyphenols, demonstrating various changes in the proteins’ structures [27]. In fact, these interactions may lead to aggregation of the proteins or conformational changes.

### 2.2. Collagen Fibrils Coatings Characterization

**Figure 2:**
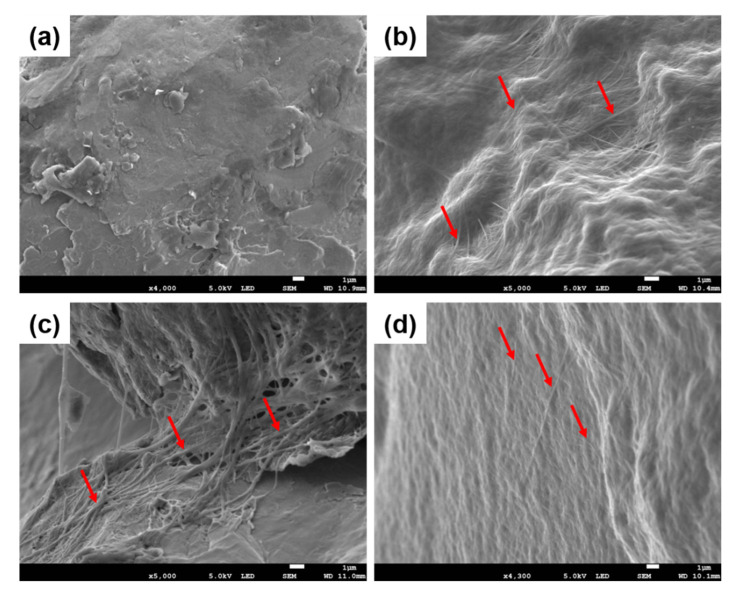
SEM images of (**a**) uncoated Ti, and (**b**) Ti_Col, (**c**) Ti_Col_low and (**d**) Ti_Col_high coatings. Ti: titanium alloy Ti6Al4V; Ti_Col: collagen-coated Ti; Ti_Col_low: collagen and low PG concentration-coated Ti; Ti_Col_high: collagen and high PG concentration-coated Ti. Red arrows indicate collagen fibrils.

Scanning Electron Microscopy (SEM) images of collagen coatings (Figure 2) confirmed the presence of collagen fibrils adsorbed on the surface of Ti6Al4V samples, showing that the fibrils were formed and remained onto the surface after washing. Collagen fibrils formed a homogeneous network on all the Ti surfaces with and without PG. At a magnification of ×15,000, the coverage of the Ti_Col_low surface by fibrils is poorer (Supplementary Figure S1). It is conceivable that the presence of PG may influence the thickness and the spatial distribution of fibrils adsorbed on the surface of Ti6Al4V.

Contact angle (CA) measurements clearly demonstrated the presence of the coating by a decrease of the contact angle from about 115° (uncoated Ti6Al4V) to 95° (Ti_Col). Moreover, when PG was added to the coatings, the CA could not be measured, since the water droplet was directly absorbed into the samples, presumably due to the high roughness of the Ti6Al4V substrate. This suggests that the presence of PG in the coatings made the coatings markedly more hydrophilic.

**Figure 3:**
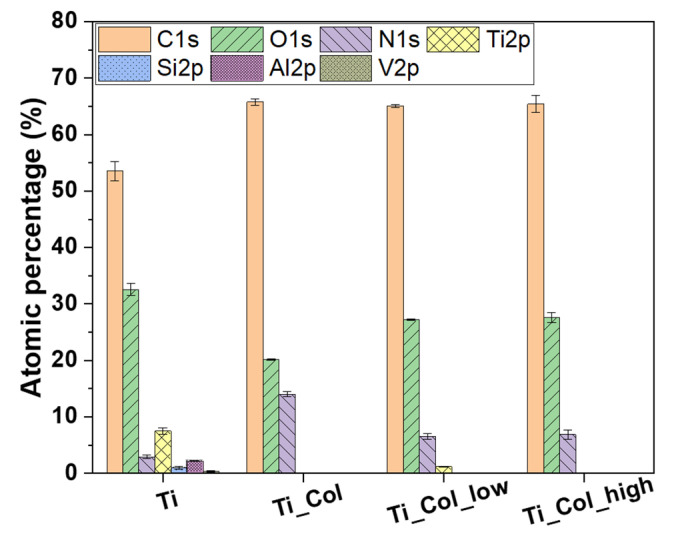
Atomic composition of uncoated and coated titanium samples. Error bars represent the standard error of the mean. Ti: titanium alloy Ti6Al4V; Ti_Col: collagen-coated Ti; Ti_Col_low: collagen and low PG concentration-coated Ti; Ti_Col_high: collagen and high PG concentration-coated Ti.

X-ray photoelectron spectroscopy (XPS) analyses revealed that the surface of Ti6Al4V consisted of C (54%), O (33%), and Ti (7%), and a small amount of N (3%), Al (2%), Si (<1%), and V (<1%) (Figure 3). This composition is similar to previous studies of others on this type of Ti6Al4V obtained by AM [28]. By the addition of the collagen coating (Ti_Col), the N1s and C1s features increased up to 14% and 66%, respectively, due to the chemical composition of proteins, which contain N and C. With the addition of PG into the coatings (Ti_Col_low and Ti_Col_high), the percentage of O increased due to the presence of O in PG. Finally, the elements of the Ti6Al4V, such as Ti and Al, were not detectable when the fibrils were adsorbed, suggesting coverage of the surface by the coating. A small amount of Ti (<2%) was still present on Ti_Col_low samples. One may speculate that the coating on this sample may be less uniform or thinner than the other coatings, although the fibrils observed on Ti_Col_low samples by SEM (Figure 2) did not appear to be significantly thinner than those on Ti_Col or Ti_Col_high samples. Alternatively, the randomly chosen analyzed spots may simply have been less well coated on the Ti_Col_low sample.

### 2.3. In Vitro Studies

#### 2.3.1. Osteogenic Differentiation

**Figure 4:**
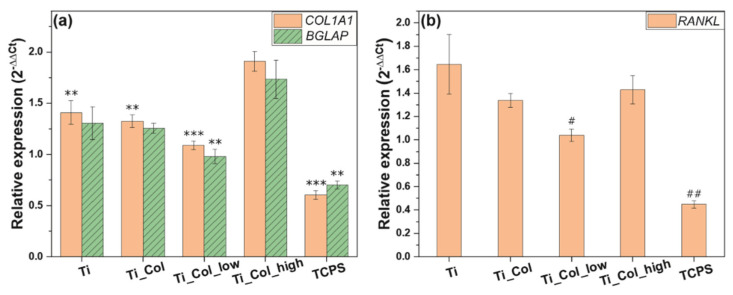
Relative expression of (**a**) *COL1A1* and *BGLAP* genes and (**b**) *RANKL* gene in osteoblast-like cells. Ti: titanium alloy Ti6Al4V; Ti_Col: collagen-coated Ti; Ti_Col_low: collagen and low PG concentration-coated Ti; Ti_Col_high: collagen and high PG concentration-coated Ti. Error bars represent the standard error of the mean. One-way ANOVA and Bonferroni corrections for multiple comparisons. A significant level of 0.05 was used throughout the study. * Significant difference between Ti_Col_high and other tested samples/Ti and Tissue Culture Polystyrene (TCPS) controls. ** *p* < 0.01; *** *p* < 0.001. # Significant difference between Ti control and tested samples/TCPS control. # *p* < 0.5, ## *p* < 0.01.

Expression of genes for osteogenic differentiation were studied using the osteoblastic cell line SaOS-2. The collagen coating with the highest PG concentration (Ti_Col_high) clearly promoted the osteogenic differentiation by an increase of the relative expression of collagen type I alpha 1 chain (*COL1A1*) and bone gamma-carboxyglutamate protein (*BGLAP*) genes from 1.41 ± 0.12 (Ti) to 1.91 ± 0.10 (Ti_Col_high) and 1.30 ± 0.16 (Ti) to 1.73 ± 0.19 (Ti_Col_high), respectively (Figure 4a). However, these relative expressions slightly decreased when there was no PG (Ti_Col) or a low concentration (Ti_Col_low) compared to uncoated samples. *COL1A1* is known as a key marker of bone matrix production, while *BGLAP* is involved in the regulation of mineralization process [29]. Thus, our results clearly demonstrated that the Ti coating with the high concentration of PG (Ti_Col_high) might stimulate both bone matrix formation and the mineralization process in early stages of osseointegration. The relative expression of the *COL1A1* and *BGLAP* genes was slightly decreased for the Ti_Col and Ti_Col_low samples, when compared to uncoated Ti surfaces. However, as shown in Figure 4a, significant differences have been observed between uncoated Ti surfaces and Ti_Col/Ti_Col_low samples. Hence, based on the significant differences, authors assumed that Ti_Col_high contributes toward the promotion of the expression of the genes *COL1A1* and *BGLAP*, while the effect of Ti_Col and Ti_Col_low on osteogenic markers expression was not significant.

Our findings are in line with other studies investigating the in vitro and in vivo effect of phenolic compounds extracted from marine green macroalgae on bone matrix mineralization process. Quantitative analysis of extracellular matrix mineralization of a fish bone-derived cell line and in zebrafish larvae stained with alizarin red S showed the osteogenic potential of phenolic compounds [30]. Our results confirmed the ability of PG to regulate bone matrix formation and the mineralization process on the gene expression levels of osteogenic markers.

During osseointegration, mineralized bone is constantly renewed by means of the bone remodeling process. This process consists of bone resorption by osteoclasts, followed by bone formation by osteoblasts [31]. The ligand for receptor activator of nuclear factor-κB (*RANKL*) is a marker of osteoclast activation and bone resorption. In our study, the relative expression of *RANKL* decreased slightly in the presence of the “collagen only” coating (Ti_Col) and the coating with a low concentration of PG (Ti_Col_low) (Figure 4b). When the PG concentration was increased to the higher level (Ti_Col_high), the relative expression seemed to increase as well. The reduction of the relative expression of *RANKL* marker is important since it shows the activation of osteoclasts indicating the initiation of remodeling process. Up-regulation of *RANKL* in the presence of Ti coating with high concentration of PG (Ti_Col_high) might be a result of a strong up-regulation of *COL1A1* and *BGLAP* genes, as shown in Figure 4a. In our previous studies investigating the in vitro effect of plant-derived polysaccharides coatings, we have shown that the expression of osteoclastogenesis marker *RANKL* corresponds with the expression of osteogenic markers (*COL1A1*, *ALPL* and *BGLAP*) in murine primary calvarial osteoblasts and the pre-osteoblast cell line MC3T3-E1 at different stages of osteoblast differentiation. Moreover the expression of *COL1A1*, *ALPL*, *BGLAP,* and *RANKL* in cultured osteoblasts was shown to increase with cell differentiation, suggesting that mature and differentiated osteoblasts support osteoclast activation through increased *RANKL* expression [32,33]. According to literature, the differentiated osteoblast cells promote osteoclastogenesis to a greater extent than undifferentiated osteoblasts [34]. However, further research is needed to investigate the effect of phenolic-enriched collagen fibrillar coatings on osteoclastogenesis and bone resorption. Additional examination of the protein known as osteoprotegerin (OPG), which is a decoy receptor for RANKL that prevents the binding of *RANKL* to *RANK*, thereby suppressing osteoclast activation, may be useful to determine the impact of PG coatings on bone resorption [35].

#### 2.3.2. Inflammatory Response

Insertion of titanium implants into the bone triggers an inflammatory response, which is important for the osseointegration process. Acute inflammation is initiated by the pro-inflammatory cytokines, including interleukin-6 and tumor necrosis factor-alpha, released by immune cells, as well as by osteoblasts and fibroblasts of surrounding tissues. However, the excessive release of pro-inflammatory cytokines indicates activation of chronic inflammation, which leads to a failure of osseointegration [36]. Therefore, the gene expression of the following pro-inflammatory cytokines was examined: interleukin-6 (*IL6*) and tumor necrosis factor-alpha (*TNFA*) in osteoblast-like cells (SaOS-2) and fibroblast-like cells (3T3 cell line).

**Figure 5:**
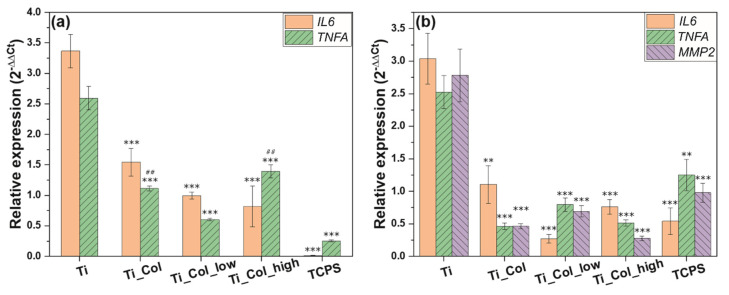
Relative expression of inflammatory markers in (**a**) osteoblast-like cells and (**b**) fibroblast-like cells. Ti: titanium alloy Ti6Al4V; Ti_Col: collagen-coated Ti; Ti_Col_low: collagen and low PG concentration-coated Ti; Ti_Col_high: collagen and high PG concentration-coated Ti. Error bars represent the standard error of the mean. One-way ANOVA and Bonferroni corrections for multiple comparisons. A significant level of 0.05 was used throughout the study. *Significant difference between Ti control and tested samples/TCPS control. ** *p* < 0.01; *** *p* < 0.001. #Significant difference between Ti_Col_low and other tested samples/Ti and TCPS controls. ## *p* < 0.01.

The expression of *IL6*, an inflammatory marker, was significantly reduced by the presence of all coatings (Figure 5). The collagen was the main influence in reducing this expression by more than half for each cell type. In fact, for osteoblast-like cells, *IL6* expression decreased from 3.36 ± 0.28 (Ti) to 1.55 ± 0.23 (Ti_Col) and *TNFA* from 2.59 ± 0.19 (Ti) to 1.11 ± 0.04 (Ti_Col) (Figure 5a). For fibroblast-like cells, *IL6* was reduced even more from 3.04 ± 0.39 (Ti) to 1.11 ± 0.29 (Ti_Col), as well as *TNFA* from 2.52 ± 0.26 (Ti) to 0.46 ± 0.05 (Ti_Col). However, PG seemed to have an impact as well, by reducing the *IL6* expression to less than 1. This decrease is most robust on the Ti_Col_low coating, while the Ti_Col_high coating decreased the expression of *TNFA*. Nevertheless, the differences in expression of pro-inflammatory markers between Ti coated with low and high concentrations of PG, i.e., Ti_Col_low and Ti_Col_high, were not significant (*p* < 0.05). The inflammatory response was clearly reduced by all tested coatings compared to the uncoated Ti6Al4V. The obtained results indicate the ability of PG coatings to reduce the gene expression of the pro-inflammatory cytokines, in particular *IL6*. In addition, down-regulation of pro-inflammatory genes in the presence of PG coatings prevents bone degradation as it has been reported that *IL6* and *TNFA* enhance osteoclast activation and bone resorption [37]. Our findings are in line with previous studies investigating the anti-inflammatory effect of PG on macrophage-like cells [15].

Bone loss leading to implant failure is triggered not only by excessive cytokine secretion, but also by release of proteolytic enzymes, mainly represented by metalloproteinase (MMP) [38]. These degrading enzymes have a pivotal role in extracellular matrix remodeling. However, the overexpression of genes encoding MMP results in rapid bone degradation [39]. The expression of *MMP2*, the gene encoding metalloproteinase-2 protein, was significantly reduced by the presence of all coatings (Figure 5b). The lowest expression of *MMP2* expression was observed on Ti coatings with high concentration of PG (Ti_Col_high). Interestingly, fibrillar coatings of collagen type I on tissue culture dishes promoted the expression of *MMP1*, while coatings of tropocollagen did not [40].

In summary, the results of gene expression indicate that Ti coatings with PG, in particular containing PG in high concentration (Ti_Col_high), seem to promote osteogenic activities while reducing inflammation in vitro.

#### 2.3.3. Cell Morphology

**Figure 6:**
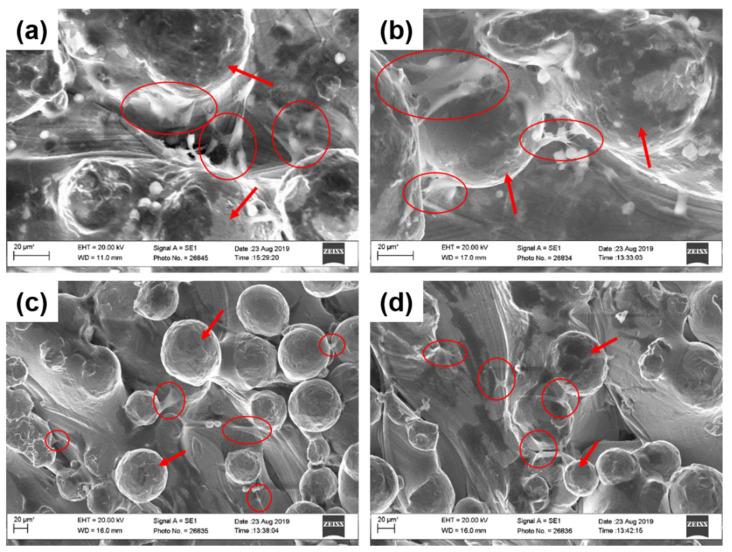
SEM images of (**a**) osteoblasts on Ti uncoated and on (**b**) Ti_Col_high coating and (**c**) fibroblasts on uncoated Ti and on (**d**) Ti_Col_high coating after 3 days of culture. Circles indicate cells and arrows indicate particles of Ti6Al4V powder (scale bars: 20 μm). Ti: titanium alloy Ti6Al4V; Ti_Col: collagen-coated Ti; Ti_Col_low: collagen and low PG concentration-coated Ti; Ti_Col_high: collagen and high PG concentration-coated Ti.

SEM images confirmed the attachment of osteoblasts and fibroblasts on uncoated and coated Ti6Al4V samples after 3 days of culture (Figure 6). Moreover, cells seemed to spread well on both samples between particles of Ti6Al4V powder. However, no significant difference in the cell morphology between the samples groups was detectable.

## 3. Materials and Methods

### 3.1. Production of Ti6Al4V Samples

Electron beam melting (EBM^®^), a powder-bed fusion technology was used to prepare the samples [41]. In this technology, the components are manufactured layer by layer in vacuum from a metallic precursor powder melted by an intense electron beam. Components were designed as 3D geometry models using standard Computer Aided Design (CAD) system. A number of different components to be simultaneously manufactured in a single batch is assembled into corresponding ‘build file’. Next, this assembly of 3D image is broken into one layer- thick “slices” and loaded into the machine for automated manufacturing. Sample scaffolds with the diameter 7 mm were fabricated in ARCAM A2 EBM^®^ machine by Arcam EBM (Göteborg, Sweden) using 50 µm layer thickness and standard process parameter settings from the manufacturer’s firmware. Ti6Al4V precursor powder was used and produced by Arcam EBM, which had close to spherical particles shape with the size distribution from 75 to 125 µm. Due to the specifics of EBM^®^ technology, after the manufacturing, the samples are embedded into the semi-sintered precursor powder. To recover the samples, they are treated in the powder recovery system (a part of the Arcam EBM^®^ machine environment), where the jet of the compressed air with precursor powder strips all particles not fused into the outer surfaces of the samples. This procedure is performed according to the standard protocol prescribed by the Arcam EBM^®^ machine manufacturer. No additional treatment to the samples was done prior to the sample coating.

### 3.2. Preparation of Collagen Hydrogels and Coatings

**Figure 7:**
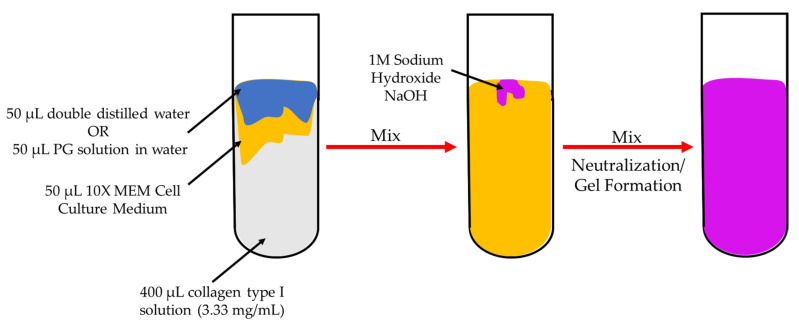
Diagram of preparation of collagen hydrogels. Collagen hydrogels were prepared by mixing 50 μL of double distilled water or 50 μL PG solution in water with 50 μL 10X MEM Cell Culture Medium and 400 μL of collagen type I solution (3.33 mg/mL). Then, with the neutralization of the solution by adding some drops of 1M NaOH solution, the solution became violet and hydrogels formed.

Collagen hydrogels were prepared following the protocol of Karamichos et al. [42], as described in previous studies [10,11] (Figure 7). Collagen hydrogels were also prepared by replacing the double distilled water with 50 μL phloroglucinol (PG) in water at a low concentration of 0.333 mg/mL and a high concentration of 1.0 mg/mL (Table 1). Then, small drops of NaOH solution were added to neutralize the solution. After addition of each small drop, the solution was shaken gently. Neutralization was deemed to have occurred when the color of the solution remained violet after shaking. All hydrogels formed within 5 min of neutralization. Hydrogels formed as a result of fibrillogenesis, leading to the formation of a 3D network of collagen fibrils. These hydrogels were placed on Ti6Al4V samples for 1 h to allow adsorption of fibrils from the hydrogel onto the surface of Ti6Al4V. Then, the substrates were washed vigorously with double-distilled water and dried under room conditions. Coatings of collagen, collagen and 0.333 mg/mL PG, and collagen and 1.0 mg/mL PG were named Ti_Col, Ti_Col_low, and Ti_Col_high, respectively (Table 2).

### 3.3. Physicochemical Characterization of the Collagen Hydrogels and Coatings

Collagen hydrogels were freeze-dried and analyzed by FTIR spectroscopy (Agilent Cary 630, Agilent Technology, Cheshire, UK) in Attenuated Total Reflectance (ATR) mode. Spectra were collected in the 500–4000 cm^−1^ spectral range with a resolution of 4 cm^−1^ and an average of 8 scans.

Collagen fibril coatings were characterized by SEM (JEOL JSM-7800F) as described previously without gold coating [10]. XPS measurements were performed on the coatings with an Axis Supra spectrometer (Kratos Analytical Ld, Manchester, UK). All spectra were analyzed with CasaXPS software version 2.3.22. Contact angle measurements were obtained with a homemade system composed of a light, a support, and a camera connected to a computer. The images were analyzed with ImageJ software.

### 3.4. In Vitro Studies

#### 3.4.1. Cell Culture

Human osteosarcoma cell line SaOS-2 and mouse embryonic fibroblast 3T3 cell line were obtained from Culture Collections, Public Health England, London. Both cell lines were grown in cell culture medium consisting of minimum essential medium (Gibco, Paisley, UK), 10% fetal bovine serum (FBS) (Invitrogen, Paisley, UK), antibiotic (100 mg/L streptomycin and 100 U/mL penicillin) (Sigma-Aldrich, Gillingham, UK), 2 mM L-glutamine (Biochrom Ltd., Cambridge, UK), and incubated at 37 °C with 5% CO_2_ (HerAcell 150, Heraeus, Hanay, Germany). For all in vitro assays, 5 × 10^4^ cells/well were cultured for 3 days on each uncoated and coated sample (Ti, Ti_Col, Ti_Col_low, Ti_Col_high) placed in 48-well tissue culture polystyrene (TCPS) plates (Life technologies, Paisley, UK). Experiments were performed in triplicate (*n* = 3). TCPS wells were used as controls in this study.

#### 3.4.2. RNA-Isolation and cDNA Reverse Transcription (RT)

To determine the expression of mRNA, total RNA was isolated after 3 days from the attached cells using TRI reagent (Sigma-Aldrich) and the RNeasy Mini Kit (Qiagen, Crawley, UK). The protocol was followed according to the manufacturer’s specification. The concentration of RNA was determined by UV spectrometry (Eppendorf, Hamburg, Germany) at 260 nm.

The RNA was reversed transcribed to cDNA using one-step high-capacity cDNA RT kit (Applied Biosystems, Warrington, UK) according to the manufacturer’s instructions.

#### 3.4.3. Real-Time Polymerase Chain Reaction (PCR)

Real-time PCR was performed on the Light Cycler 480 instrument (Roche Diagnostics GmbH, Mannheim, Germany) using Roche SYBR Green PCR Master Mix in duplicates according to the manufacturer’s instructions. The primer sequences (Sigma-Aldrich, Gillingham, UK) for the specific target genes are presented in Table 3. *ACTB* and *Gapdh* were used as housekeeping genes. Real-time PCR reactions were carried out in 10 μL volumes in a 96-well plate (Roche Diagnostics GmbH, Mannheim, Germany) containing 1 μL of cDNA and 9 μL reaction mixture, according to the manufacturer’s instructions. PCR conditions consisted of an initial denaturation step of 95 °C for 5 min, followed by 40 cycles of 95 °C for 10 s, 60 °C for 15 s, and 72 °C for 20 s. The comparative 2^−ΔΔCt^ method was performed for analysis of relative gene expression data, as previously described by Livak et al. [43]. Relative expression levels were calculated for each sample after normalization against the housekeeping gene.

#### 3.4.4. Statistical Analyses

Data are presented as the mean values ± standard error of the mean. Significant differences in the in vitro studies were tested using one-way ANOVA, followed by a multiple comparison Bonferroni test using SPSS version 22 (IBM, Armonk, NY, USA). A *p* value 0.05 was considered significant.

## 4. Conclusions and Outlook

To conclude, FTIR spectra demonstrated that hydrogels were made of collagen and PG affected the hydrogels by wavelength shifts. Furthermore, the XPS results revealed the presence of the coatings after adsorption of fibrils from the hydrogels, after washing and drying. Hence, the coatings are stable and PG is present. The CA measurements showed an increase of the hydrophilicity with coated samples.

Moreover, fibroblast-like cells and osteoblast-like cells seemed to attach and spread well on the coatings. In vitro experiments demonstrated that PG-enriched coatings significantly reduced the gene expression of inflammation markers. Moreover, the expression of markers of osteogenic differentiation may be promoted with a high PG concentration and the expression of the marker of osteoclast activation may be reduced with a low PG concentration.

Thus, PG-enriched collagen fibril coatings could constitute a promising approach to improve implants for bone reconstruction, with the aim of promoting bone formation at the implant surface by stimulating osteogenic differentiation while inhibiting inflammation. Having established the effects of coatings and PG on gene expression of relevant markers for inflammation and osteogenic differentiation, future work should focus on detection of proteins characteristic of osteogenic differentiation such as alkaline phosphatase (ALP) and mineral formation and in vivo experiments to test the effect of coatings on osseointegration. Based on our previous experiments with primary osteoblasts isolated from mice and osteoblastic cells MC3T3-E1, the long-term matrix deposition (Alizarin Red-S) was correlated with the time of osteogenic markers expression (*RUNX-2*, *COL1A1*, *ALPL*, *BGLAP*) [32,33]. The studies were performed on plant-derived coating and similar experiments should be performed on Ti6Al4V coated with collagen to confirm the correlation.

## Figures and Tables

**Table 1:** Collagen hydrogels composition.

Solution	Collagen Type I Solution (3.33 mg/mL)	Double-Distilled Water	PG Solution (in Water)
1	400 μL	50 μL	None
2	400 μL	None	50 μL (0.333 mg/mL)
3	400 μL	None	50 μL (1.0 mg/mL)

**Table 2:** Coatings denomination.

	Coating Type
Ti	No coating
Ti_Col_low	Collagen hydrogel containing low PG concentration
Ti_Col_high	Collagen hydrogel containing high PG concentration

**Table 3:** Primer sequences for Real-Time PCR.

Origin	Gene Name	Gene Abbreviation	Primer	Sequence 5′ to 3′
Mouse	glyceraldehyde-3-Phosphate Dehydrogenase	*Gapdh*	Forward	CCCATCACCATCTTCCAGGAGC
			Reverse	CCAGTGAGCTTCCCGTTCAGC
	interleukin6	*Il6*	Forward	GAGGATACCACTCCCAACAGACC
			Reverse	AAGTGCATCATCGTTGTTCATACA
	tumor necrosis factor-alpha	*Tnfa*	Forward	GATCTCAAAGACAACCAACATGTG
			Reverse	CTCCAGCTGGAAGACTCCTCCCAG
	matrix metalloproteinase 2	*Mmp2*	Forward	AAGGATGGACTCCTGGCACATGCCTTT
			Reverse	ACCTGTGGGCTTGTCACGTGGTGT
Human	beta-actin	*ACTB*	Forward	CACCAACTGGGACGACAT
			Reverse	ACAGCCTGGATAGCAACG
	collagen 1 type 1 alpha	*COL1A1*	Forward	GGTCAAGATGGTCGCCCC
			Reverse	GGAACACCTCGCTCTCCAG
	bone gamma-carboxyglutamate protein (osteocalcin)	*BGLAP*	Forward	CGCTACCTGTATCAATGGCTGG
			Reverse	CTCCTGAAAGCCGATGTGGTCA
	receptor activator for nuclear factor κ B ligand	*RANKL*	Forward	ACATATCGTTGGATCACAGCACAT
			Reverse	CAAAAGGCTGAGCTTCAAGCTT
	Interleukin-6	*IL6*	Forward	TGTGAAAGCAGCAAAGAGGC
			Reverse	TGATTTTCACCAGGCAAGTCTC
	tumor necrosis factor-alpha	*TNFA*	Forward	ATCCTGGGGGACCCAATGTA
			Reverse	AAAAGAAGGCACAGAGGCCA

## Supplementary Materials

The following are available online at https://www.mdpi.com/1422-0067/21/17/6406/s1, Figure S1: SEM images of (a) Ti_Col, (b) Ti_Col_low and (c) Ti_Col_high coatings.

## Abbreviations

AM	Additive manufacturing
BGLAP	Bone gamma-carboxyglutamate protein (osteocalcin)
CA	Contact angle
CAD	Computer aided design
COL1A1	Collagen type I alpha 1 chain
FTIR	Fourier transform infrared
IL6	Interleukin-6
MMP	Matrix metalloproteinases
MRSA	Methicillin-resistant *S.aureus*
OPG	Osteoprotegerin
PCR	Polymerase chain reaction
PG	Phloroglucinol
RANKL	Receptor activator of nuclear factor-κB
SEM	Scanning electron microscopy
TCPS	Tissue culture polystyrene
TNFA	Tumor necrosis factor-alpha
XPS	X-ray photoelectron spectroscopy
