# Supplementary material for: Phenolic-Enriched Collagen Fibrillar Coatings on Titanium Alloy to Promote Osteogenic Differentiation and Reduce Inflammation
Source: Int J Mol Sci. 2020 Sep 3;21(17):6406. doi: 10.3390/ijms21176406 (PMC7504673; doi:10.3390/ijms21176406)
Supplement: Supplementary file 1 [file ijms-21-06406-s001.pdf]

Supplementary data

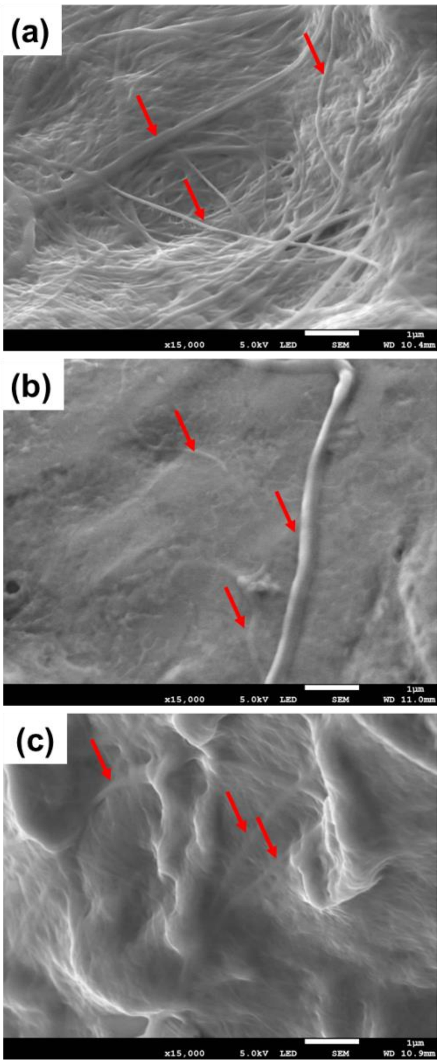

Figure S1. SEM images of (a) Ti_Col, (b) Ti_Col_low and (c) Ti_Col_high coatings. Ti: titanium alloy Ti6Al4V; Ti_Col: collagen-coated Ti; Ti_Col_low: collagen and low PG concentration-coated Ti; Ti_Col_high: collagen and high PG concentration-coated Ti (magnification: x15000; scale bar: 1 μm). Red arrows indicate collagen fibrils.
